# The Unlucky Variant: Artery of Percheron Infarction

**DOI:** 10.7759/cureus.84582

**Published:** 2025-05-21

**Authors:** James C Arcidiacono, Allyson Whitsett, Kevin Callagy, Robert Colella

**Affiliations:** 1 Internal Medicine, Jersey Shore University Medical Center, Neptune, USA; 2 Medical School, St. George's University School of Medicine, True Blue, GRD

**Keywords:** artery of percheron, cardioembolic, cardiology, neurology, stroke, thalamic ischemia

## Abstract

This case highlights the clinical significance of a rare ischemic stroke due to an artery of Percheron (AOP) infarction. If the AOP becomes blocked, it can lead to an infarct in the paramedian thalami and mesencephalon. AOP infarcts typically present with bilateral thalamic ischemia, a condition that can be challenging to diagnose due to its rarity and nonspecific symptoms. These infarcts are extremely rare, comprising only 0.1-2% of all ischemic strokes, and are often underdiagnosed. In this case, the patient presented with obtundation and later developed third cranial nerve palsy and diplopia, underscoring the need for heightened clinical awareness of atypical stroke presentations. A 69-year-old female with a past medical history significant for Ménière’s disease (included due to initial overlap with altered sensorium or vertiginous presentation), hyperlipidemia, and bradycardia, who presented with obtundation. Initial CT imaging and video electroencephalogram were unremarkable. The patient’s mental status improved over time, but she subsequently developed a third cranial nerve palsy and diplopia. An MRI brain revealed bilateral thalamic diffusion restriction and edema without enhancement, consistent with bilateral thalamic ischemic infarcts in the AOP territory. The patient was started on dual antiplatelet therapy (DAPT) for 21 days for secondary stroke prevention. A CT venogram (CTV) was ordered to rule out venous sinus thrombosis (VST). Additionally, an implantable loop recorder (ILR) was placed to monitor for potential atrial fibrillation as a possible source of cardioembolic stroke during outpatient follow-up. This case highlights the complexity of diagnosing and managing AOP infarction, a rare type of stroke.

## Introduction

The blood supply to the thalami and midbrain comes from small branches of the posterior cerebral and communicating arteries. The thalamus itself receives blood in four main regions: anterior, paramedian, posterior, and inferolateral. The paramedian region is nourished by small arteries branching off from the posterior circulation, known as the paramedian arteries. There are four common patterns of blood supply to the thalami and midbrain. In the most frequent pattern, called Variant I, the paramedian arteries originate separately from the right and left posterior cerebral arteries (PCAs) [1]. Variant IIa involves both paramedian arteries coming from the left P1 segment, while in Variant IIb, the artery of Percheron (AOP) arises from the P1 segment of the PCA and supplies both paramedian thalami and the upper midbrain. Variant III, known as the arcade variant, has a network of small branches forming an arc between the P1 segments and the PCAs [1].

If the AOP becomes blocked, it can lead to an infarct in the paramedian thalami and mesencephalon. The AOP variant anatomy is seen in about 4%-12% of patients, and large studies of stroke cases have shown that infarcts involving the AOP make up only 0.1%-2% of all ischemic strokes, highlighting its rare occurrence [1].

This case highlights the clinical significance of a rare ischemic stroke due to an AOP infarction. To define strokes, we separate them largely into two categories: ischemic vs hemorrhagic. Ischemic involves a thromboemboli blocking blood flow through the vessel, leading to downstream ischemia in the brain. Hemorrhagic strokes normally present as an intracerebral hemorrhage, representing 10% of all strokes while 87% of all strokes are of the ischemic type [2].

AOP infarcts typically present with bilateral thalamic ischemia, a condition that can be challenging to diagnose due to its rarity and nonspecific symptoms. These often present with vague symptoms such as reduced consciousness, cognitive changes or confusion without obvious focal deficits. These rare strokes are at risk of being missed in the initial assessment as bilateral thalamic infarction is often misdiagnosed, diagnosed late, or even not considered at all because of the wide variety of presentations. In this case, the patient presented with obtundation and later developed third cranial nerve palsy and diplopia, underscoring the need for heightened clinical awareness of atypical stroke presentations. The cryptogenic nature of the infarct, with concerns for underlying cardioembolic sources such as undiagnosed atrial fibrillation (AF) or venous sinus thrombosis, highlights the importance of comprehensive diagnostic evaluations. By employing advanced diagnostic techniques such as CT venography and implantable loop recorders, this case exemplifies a modern, multidisciplinary approach to managing rare and complex stroke cases, making it a valuable contribution to the medical literature.

## Case presentation

The patient is a 69-year-old female with a past medical history significant for Ménière's disease, hyperlipidemia, and bradycardia, who presented with altered mental status, specifically obtundation. She was last seen at her baseline the previous night after returning from a social event. Around 6:30 AM, her husband noted difficulty waking her, accompanied by heavy snoring and a diminished response to stimuli, although she was normally reactive. On initial examination, the patient was stuporous. She localized pain with her upper extremities bilaterally and moved all extremities spontaneously but did not open her eyes to command. When stimulated with painful stimuli, she would state her name but would not respond to verbal commands. Narcan was administered twice with no improvement. The patient was afebrile with a heart rate of 60 beats per minute, respiratory rate of 18 breaths per minute, blood pressure of 152/75mmHg, and oxygen saturation of 96% on room air. The patient's laboratory findings were largely unremarkable. Her blood alcohol concentration was minimal (<0.003), and her venous blood gas showed a mild respiratory acidosis with a pH of 7.313 and a pCO2 of 53.9. Blood glucose was within normal limits at 99 mg/dL, and her anion gap was 5, indicating no significant metabolic derangement. Acetaminophen and salicylate levels were negligible (<2 and <3, respectively), and the urine drug screen was negative for substances of abuse.

**Figure 1:**
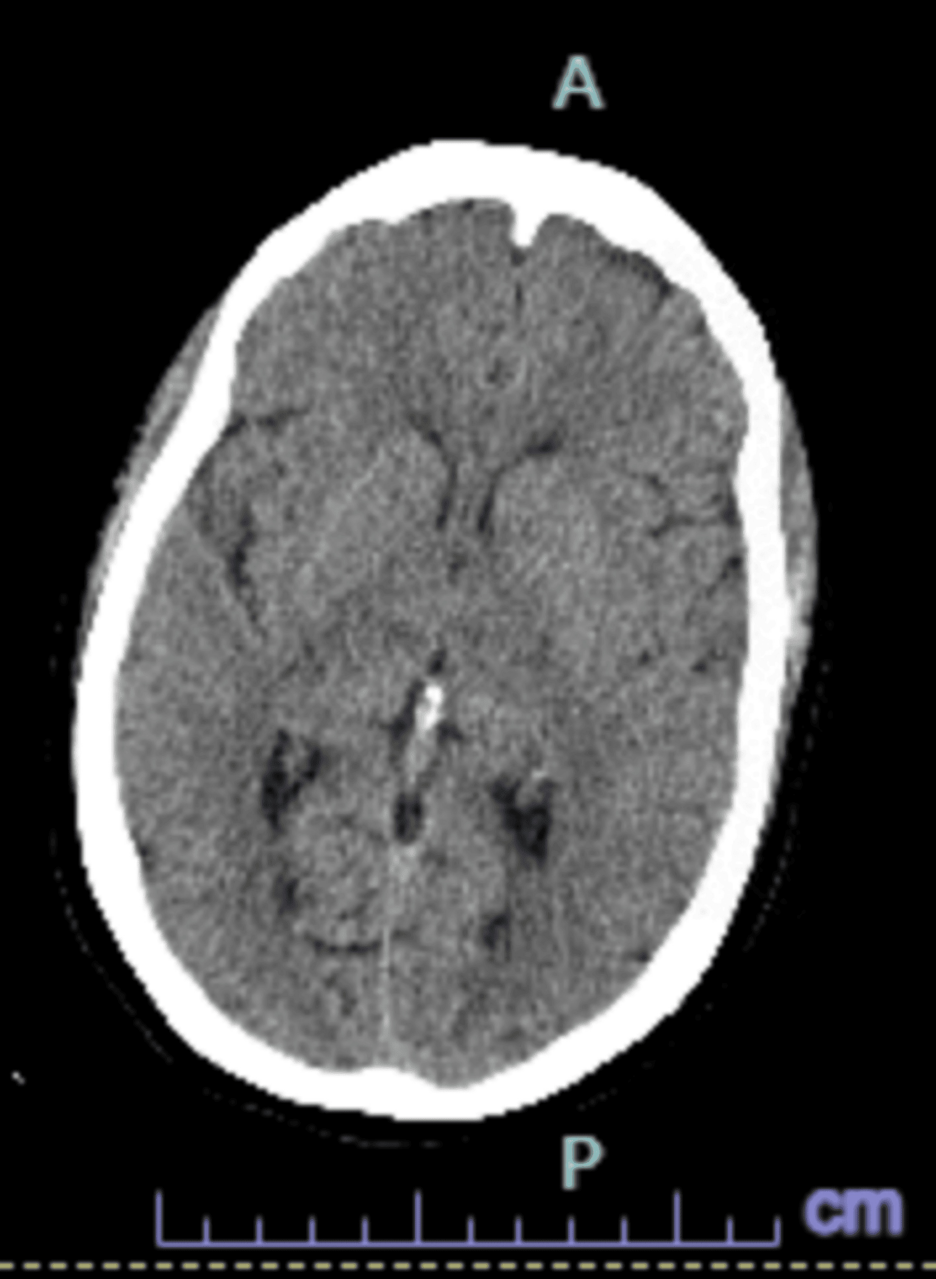
CT Code Stroke Head Without Contrast. Axial View. Taken at the time of the Code Stroke. This was negative for acute infarct, hemorrhage, or midline shift, demonstrating why CT, while abundantly used, is not a good method to detect early infarction. CT: computed tomography

**Figure 2:**
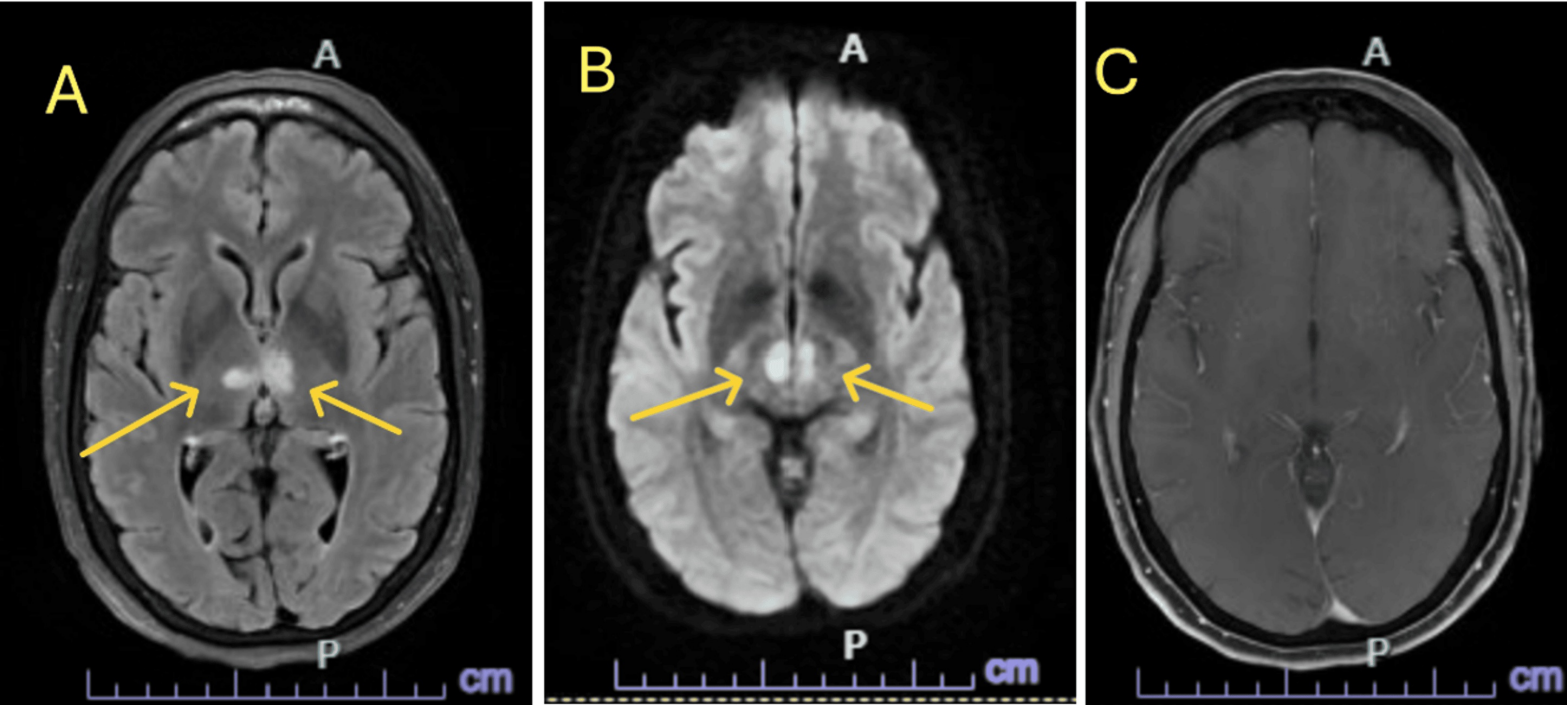
MRI Brain axial view. T2 FLAIR Propeller (A). DWI (B). T1 SPGR FS +C (C). This image shows bilateral medial thalamic diffusion restriction and edema without associated enhancement, consistent with AOP territory infarction and raised this differential diagnosis to the top position. Cytotoxic edema, characterized by restricted diffusion, reflects swelling of individual cells due to impaired cellular function. MRI: magnetic resonance imaging, Ax: axial, T1: spin-lattice relaxation time, SPGR: spoiled gradient recalled, FS: fat saturation, +C: with contrast, FLAIR: fluid-attenuated inversion recovery, DWI: diffusion-weighted imaging, AOP: artery of Percheron

A Code Neuro was initiated. A non-contrast CT of the head was negative for acute findings (Figure 1). The patient was not a candidate for tenecteplase (TNK) due to the unclear time of symptom onset, as she was last seen normal the night before. This placed her outside the window for safe thrombolysis. She was admitted to the ICU for suspected seizure activity. Continuous video EEG (VEEG) showed no evidence of seizures. A CT angiogram revealed no large vessel aneurysm, occlusion, or high-grade stenosis. CT perfusion did not show core infarcts or penumbral tissue. An echocardiogram demonstrated normal ejection fraction. The patient’s mental status improved over time, but on hospital day two, she subsequently developed a third cranial nerve palsy and diplopia. Given these findings, the stroke team was consulted and an MRI of the brain was ordered. MRI findings revealed bilateral thalamic diffusion restriction and edema without enhancement, consistent with bilateral thalamic ischemic infarcts in the AOP territory (Figure 2). The etiology was cryptogenic, with concern for a cardioembolic source such as undiagnosed atrial fibrillation or deep venous sinus thrombosis (VST).

The patient was started on dual antiplatelet therapy (DAPT) for 21 days and then continued low-dose aspirin (81 mg) for secondary stroke prevention. A CT venogram (CTV) was ordered on hospital day three to rule out VST due to the bilateral nature of the thalamic infarcts, which raised concern for venous congestion despite the absence of signs of raised intracranial pressure. Additionally, an implantable loop recorder (ILR) was placed to monitor for potential atrial fibrillation as a possible source of cardioembolic stroke during outpatient follow-up. 

Infectious disease was consulted, with encephalitis deemed unlikely. An lumbar puncture was considered if no other diagnosis was reached. A CT venogram was negative for venous clot, and an ILR was placed by electrophysiology. Although a cardioembolic source such as paroxysmal AF was suspected, anticoagulation was not initiated due to lack of confirmatory findings. The team opted to await ILR results before starting anticoagulation, in line with current stroke management guidelines. The patient’s condition improved, and she was deemed stable for discharge. At discharge, the patient had partial resolution of third nerve palsy and was ambulating independently with mild cognitive slowing. She was deemed functionally stable with a good prognosis. Close outpatient follow-up was arranged, including monitoring with the ILR and further assessment by neurology and cardiology. The patient’s care team discussed alarm symptoms and risk prevention, as well as long term health goals and avoidance of recurrence.

## Discussion

The patient’s altered mental status prompted a broad differential, including metabolic, toxic, infectious, and neurological causes. The unremarkable lab findings and negative toxicology screen helped narrow the focus to a potential neurological or cardiovascular etiology. Bilateral thalamic ischemic infarcts in the AOP territory are rare and difficult to diagnose. These infarcts can present with altered consciousness, vertical gaze palsy, memory deficits, or, as in this case, third cranial nerve palsy. 

Initial considerations in the differential included toxic/metabolic encephalopathy, non-convulsive status epilepticus, brainstem encephalitis, demyelinating processes, and basilar artery thrombosis. These were systematically ruled out via normal labs, a negative EEG, lack of infectious markers, and absence of focal vascular lesions on CTA.

**Figure 3:**
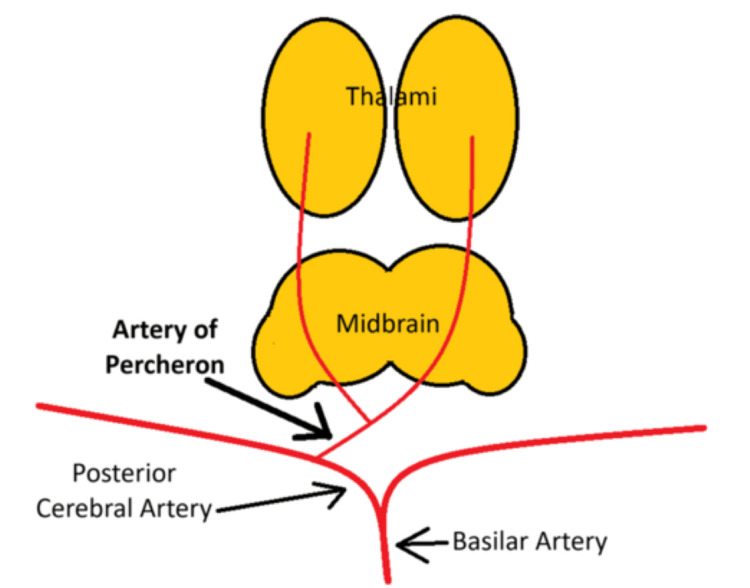
Cartoon image of Variant IIb of blood supply to the thalami and midbrain via the artery of Percheron. An infarction of this artery would cause bilateral thalamic ischemia. Image credit: James Arcidiacono

The thalamus is highly susceptible to a wide range of clinical symptoms when affected by an ischemic stroke due to its complex anatomy and varied blood supply. When the artery of Percheron, which supplies both paramedian thalami, is involved, patients typically experience three primary symptoms: vertical gaze palsy, memory impairment, and coma [3]. These symptoms are common in cases of bilateral paramedian thalamic strokes (Figure 3). Additionally, strokes in this region often extend to the midbrain, leading to a condition called "mesencephalothalamic" or "thalamopeduncular" syndrome, further expanding the clinical spectrum [3]. The etiology was likely cryptogenic but with concern for a cardioembolic source, potentially atrial fibrillation or venous sinus thrombosis. Atrial fibrillation, even if paroxysmal, can result in embolic strokes. The implantable loop recorder is an important tool for detecting such paroxysmal AF.

AOP infarcts can be difficult to detect early on CT or even MRI, and may initially be overlooked due to their non-focal, bilateral nature. Diagnosing AOP infarcts on imaging normally will happen after a hyperacute phase (first 24 hours after onset) of cerebral injury [4]. This was the case with our patient as the initial symptoms had improved but she developed new third cranial nerve palsy and diplopia. The thalamus and midbrain normally won’t be visualized on the MRI until after the hyperacute phase is complete, making diagnostic timing difficult. Early recognition is essential to initiating appropriate clinical treatment, and in this case, MRI was crucial in identifying the bilateral thalamic infarcts within the perfect window of time for diagnosis and prompt treatment. Imaging is only one of many challenges in making an AOP infarct diagnosis. Any pathology affecting areas of the thalamus and midbrain regions around the AOP may lead to a similar clinical presentation, but different etiology. These include Basilar syndrome, infections, demyelination, spongiform encephalopathy, thiamine deficiency or hypoxic injury [4]. Seizure activity, infection (e.g., encephalitis), and metabolic derangements were all considered but effectively ruled out through EEG, lab results, and infectious disease consultation.

Starting the patient on DAPT for 21 days and transitioning to low-dose aspirin for long-term stroke prevention aligns with secondary stroke prevention guidelines, particularly for cryptogenic strokes [5]. This decision aligns with the CHANCE and POINT trials, which recommend short-term DAPT in cryptogenic or minor ischemic strokes to reduce early recurrence risk [6,7]. The ILR was appropriate given the suspicion for paroxysmal atrial fibrillation, which may not have been detected on initial monitoring. A retrospective cohort study of 48,901 patients found that use of ILRs after ischemic or cryptogenic stroke led to greater detection of AF and prescription of oral anticoagulation compared with long-term continuous external monitors, although there was no statistically significant difference in mortality compared with long-term continuous external monitors [8]. Despite negative CT venogram results, ruling out VST was an essential part of the diagnostic process due to the bilateral nature of the infarct, which can mimic patterns seen in venous congestion or thrombosis.

The patient's improvement over time and discharge indicate a positive recovery trajectory, though long-term follow-up is crucial given the risk of recurrent strokes. Outpatient follow-up with neurology and cardiology, along with the ILR for AF detection, is necessary to mitigate future stroke risk and manage any potential cardioembolic source.

## Conclusions

This case highlights the complexity of diagnosing and managing bilateral thalamic ischemic infarcts in the AOP territory, a rare and often cryptogenic stroke etiology. The patient's presentation of obtundation with subsequent development of cranial nerve palsy required a broad differential diagnosis and the use of advanced imaging to reveal the underlying bilateral thalamic infarcts. Despite an initially unremarkable workup, the cryptogenic nature of the infarcts raised concerns for a cardioembolic source, warranting the use of an ILR for atrial fibrillation monitoring and DAPT for stroke prevention.

This case underscores the importance of considering rare vascular syndromes such as AOP infarction in patients with atypical neurological presentations and highlights the role of early, appropriate imaging (MRI) in preventing delayed diagnosis. Keeping a thalamic infarct high on the differential list for patients has important treatment implications. Considering the challenges of timing for diagnosis through imaging, timing is important as well for delivery of fibrinolytic therapy within a few hours of onset. Timely initiation of secondary stroke prevention strategies, including monitoring for atrial fibrillation and long-term antiplatelet therapy, remains essential in reducing the risk of recurrence. Multidisciplinary follow-up, including neurology and cardiology, is crucial for comprehensive management and optimizing patient outcomes.

The patient’s functional status at discharge showed mild residual deficits, particularly in cognition and ocular motor function, with favorable overall prognosis.
